# Polymorphism and association of growth hormone gene with growth traits in Sirohi and Barbari breeds of goat

**DOI:** 10.14202/vetworld.2015.382-387

**Published:** 2015-03-23

**Authors:** Praduman Pal Singh, Sateyndra Singh Tomar, Mohan Singh Thakur, Amit Kumar

**Affiliations:** 1 Department of Animal Genetics and Breeding, Veterinary College, Nanji Deshmukh Veterinary Science University, Jabalpur, Madhya Pradesh, India; 2 Department of Animal Genetics and Breeding, Veterinary College, Nanji Deshmukh Veterinary Science University, Mhow, Madhya Pradesh, India

**Keywords:** Barbari, growth hormone gene, goat, polymorphism, *polymerase chain reaction*-restriction fragment length polymorphism, Sirohi

## Abstract

**Aim:**

The aim was to study the polymorphism of exon 2 and exon 3 of growth hormone (GH) gene, to test the polymorphic variants for Hardy–Weinberg equilibrium and to investigate association of these polymorphisms with chest girth and paunch girth in Sirohi and Barbari breeds of goat.

**Materials and Methods:**

A total of 80 kids involving forty each of Sirohi and Barbari breeds of goat were included in the study. A good quality genomic DNA isolated from the whole blood using standard protocol were used for *polymerase chain reaction* (PCR) amplification and products obtained on restriction digestion of amplicon with enzyme HaeIII were separated on 2% agarose gel, and documented in a gel doc system. The chest girth and paunch girth of kids at birth and weekly intervals up to 4 weeks of age and subsequently at 2 months, 3 months and 6 months of age were recorded. Allele frequency and genotype distribution of polymorphism were tested for Hardy–Weinberg equilibrium by program me Genepop package. Association between different genetic variants on chest girth and paunch girth were analyzed by least squares analysis employing suitable statistical model.

**Results:**

The PCR product of genomic DNA isolated from kids of Sirohi and Barbari breeds of goat on digestion with the restriction enzyme HaeIII revealed two genotypic variants *viz*., AB and BB. None of the two breeds was in Hardy–Weinberg equilibrium for these variants. The least squares analysis of variance revealed non-significant effect of GH genotype and breed × genotype interaction on chest girth and paunch girth from birth to 180 days of age. The effect of breed was highly significant (p<0.01) at all ages.

**Conclusion:**

The present study showed that both the breeds were polymorphic at the exon 2 and exon 3 loci of GH gene under study with respect to HaeIII restriction endonuclease. None of the breeds was in Hardy–Weinberg equilibrium for this region of GH gene. In the present study, no significant association between GH genotype and chest girth and paunch girth could be established but comparatively higher chest girth and paunch girth were observed for AB genotype across the breeds.

## Introduction

Goat is the one of the principal meat producing animals in India and has immense contribution to the resource poor section of society for their livelihood. Animal exhibiting high genetic merit in growth and body measurements receive high priority in breeding programmers for meat purpose. Although, lot of progress has been achieved in animal improvement using conventional breeding methods, environmental influences limits accuracy of such methods for improving polygenic traits [1] like body measurements. However, the genetic improvement of such traits can be enhanced by marker assisted selection, which is highly accurate in estimating breeding value of animals [2]. In view of the pivotal role of growth hormone in animal growth and development, GH gene may be used as a candidate gene [3] for studying its polymorphism and association in relation to growth. On the basis of studies on a restriction fragment length polymorphism (RFLP), [4] reported that there are two alleles at the GH gene locus in sheep and goats. In sheep, in one allele (Gh1), the GH gene is represented by a single copy (GH1 gene), while in the other (Gh2) the GH gene is duplicated (GH2-N (5*) and GH2-Z (3*) genes). Restriction maps of the sheep Gh1 and Gh2 loci indicated that the GH1, GH2-N, and GH2-Z genes are all very similar.

However, the situation in goat is less clear. The sequence of goat pituitary GH cDNA was reported by [5] and [6] and the GH gene sequence was described by [7]. The coding sequence predicted from the gene sequence corresponds to the goat GH cDNA sequence and this gene was subsequently shown to be the gGH1 gene. Sequence information for the g GH2 and gGH3 genes (i.e. The duplicate genes in the second allele) is not yet available.

The present study was undertaken to identify GH gene polymorphism and to investigate the association of these polymorphisms with chest girth and paunch girth in two important breeds of goat, Sirohi and Barbari.

## Materials and Methods

### Ethical approval

The present work was carried out in accordance with the guidelines laid down by the Institute Animal Ethics Committee for the use of animal subjects.

### Experimental animals and genomic DNA isolation

A total of 80 kids involving forty each of Sirohi and Barbari breeds of goat maintained at Goat Rearing Farm, College of Veterinary Science and Animal Husbandry, Nanaji Deshmukh Veterinary Science University Jabalpur. The blood samples (5-8 ml) were collected from these animals by jugular vein puncture in a ethylenediaminetetraacetic acid coated vacutainer tube and stored at 4°C till processed. Genomic DNA was isolated from the whole blood using standard protocol [8] with slight modifications. Quantity and quality check of DNA samples were made using 0.8% agarose and Nanodrop spectrophotometer and only the samples of good quality were used for *polymerase chain reaction (PCR)* amplification.

### PCR amplification and restriction digestion

Based on the published primer by [9], the following forward and reverse primers were used for PCR amplification of the exon 2 and exon 3 region of GH gene.

Forward ‘5’-CTCTGCCTGCCCTGGACT-3’

Reverse 5’-GGAGAAGCAGAAGGCAACC-3’

The 25 μl PCR reaction mixture consisted of 3 μl (90 n g) template DNA, 1 μl of each forward and reverse primer, 12.5 μl of 2 × PCR master mix (Fermentas) and 7.5 μl of deionized water (DNAse free water). The PCR protocol comprised of an initial denaturation for10 min at 94°C followed by 30 cycles of denaturation for 1 min at 94°C, annealing for 1 min at 54°C and extension for 1 min at 72°C and final extension at 72°C for 10 min. The PCR product was analyzed electrophoretically in 2% agarose gel. Restriction digestion of amplicon was conducted in a total volume of 30 μl reaction mixture having 10 × buffer tango 2 μl, PCR reaction mixture 10 μl, restriction enzyme HaeIII (10 units/μl) 1 μl and 17 μl nuclease free water. The digested products were separated on 2% agarose gel, and images were documented in a gel doc system (Bio-Rad USA) for analysis.

### Data collection and statistical analysis

The chest girth and paunch girth of kids at birth and weekly intervals up to 4 weeks of age and subsequently at 2 months, 3 months and 6 months of age were recorded. Allele frequency and genotype distribution of polymorphism were tested for Hardy–Weinberg equilibrium by program Genepop package [10]. Association between different genetic variants on chest girth and paunch girth were analyzed by least squares analysis using “mixed model least squares and maximum likelihood computer program PC-2” [11] employing the following statistical model.

Y_ijk_ = µ + a_i_ + B_j_ + (aB) _ij_ + e _ijk_

Where, Y_ijk_ is the observation of trait on k^th^ goat of the j^th^ breed and i^th^ genotype. µ is the overall mean. A_i_ is set of random cross-classified effects due to genotypes. B_j_ is set of fixed effects due to breeds. (aB) _ij_ is interaction between i^th^ genotype and j^th^ breed. e_ijk_ is a random error.

## Results and Discussion

**Figure-1:**
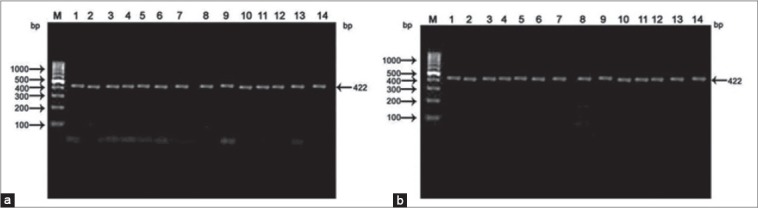
(a) Amplified polymerase chain reaction product of Sirohi electrophoresed on 2% agarose. M: 100 bp DNA ladder, Lanes: 1-14 are amplified polymerase chain reaction product. b: Amplified polymerase chain reaction product of Barbari electrophoresed on 2% agarose. M: 100 bp DNA ladder, Lanes: 1-14 are amplified polymerase chain reaction product.

An amplified PCR product of 422 bp size was observed in both the breeds studied i.e. Sirohi and Barbari on amplification of exon 2 and exon 3 of GH gene (Figure-1a and b). The PCR product of similar bp size has also been reported by [9,12] in Boer bucks and [13] in Egyptian and Saudi breeds of goat (Barki, Zaribi, Ardi and Masri) by amplification of these exons of the GH gene.

**Figure-2:**
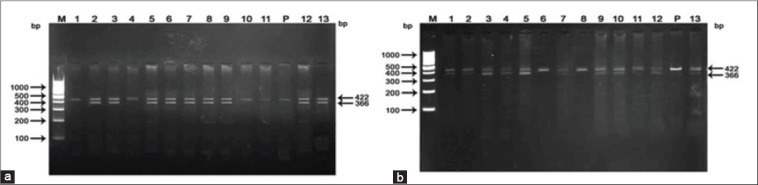
a: Polymerase chain reaction-Restriction fragment length polymorphisms pattern of growth hormone gene digested with HaeIII in Sirohi. M: 100 bp DNA ladder, P: Polymerase chain reaction product (422 bp), Lanes: 2-3,5-9,12-13 AB genotype, Lanes: 1,4,10-11 BB genotype, small fragment of 56 bp was invisible in the gel. b: Polymerase chain reaction-Restriction fragment length polymorphisms pattern of growth hormone gene digested with HaeIII in Barbari. M: 100 bp DNA ladder, P: Polymerase chain reaction product (422 bp), Lanes: 1-5, 7, 9-10, 12-13 AB genotype, Lanes: 6, 8, 11 BB genotype, small fragment of 56 bp was invisible in the gel.

Digestion of PCR product with restriction endonuclease, HaeIII revealed two band patterns (Figure-2a and b) in both the breeds indicating that the GH gene region under study was polymorphic for restriction enzyme HaeIII in these breeds. Genotyping of the polymorphic variants were done in accordance with the method employed by [9] in Boer goats. The patterns evolved in the present study showed that the presence of one restriction site on one allele and absence of restriction sites on other allele resulted in the appearance of three bands of 422, 366, and 56 bp. This genotype was referred to as AB. The 56 bp was invisible on agarose gel due to its small size. However, the absence of restriction sites on both the alleles resulted in the appearance of only one band of 422 bp. This genotype was referred to as BB. The HaeIII digestion patterns of amplicons obtained in the present study is in congruence with the findings of [9,12] in Boer goats and [13] in Egyptians and Saudi goat breeds.

### Genotypic and allelic frequency

The genotypic frequencies of AB and BB were found to be 0.82 and 0.18 in Sirohi and 0.90 and 0.10 in Barbari goats, respectively. The respective allelic frequencies of A and B were 0.41 and 0.59 in Sirohi and 0.45 and 0.55 in Barbari. None of the two breeds was in Hardy–Weinberg equilibrium for these variants. The frequency of AB genotype obtained in the present study is in consonance with the findings of [9] who reported genotypic frequency of AB as 0.837 in Boer bucks. However, they reported the presence of genotypes AA and AB; Genotype BB was missing in the sample of goats studied by them. This inconsistency may be due to breed difference and may also be the consequence of sampling fluctuations of populations under study. The estimates of allelic frequencies obtained in the present study are close to those reported by [13] in Barki goat breed, their values being 0.41 and 0.59 for allele A, and B, respectively. However, [9] in Boer goat breed and [13] in Zaribi goat breed reported allelic frequency for A to be 0.5812, and 0.620 and that for B to be 0.4188, and 0.380, respectively. These differences in allelic frequencies might be due to the fact that the different breeds/populations maintained under the different sets of environmental conditions are subject to different evolutionary forces to varying degree. In addition, sampling fluctuations might also have contributed to the differences in allelic frequencies in different breeds and populations.

### Association of GH gene polymorphism with chest and paunch girth

The least squares analysis of variance (Table-1) employed to analyze association of polymorphic variants of GH gene with chest girth at birth to 180 days of age revealed highly significant effect (p<0.01) of breed on chest girth at all stages. The effect of other factors i.e. genotype and breed × genotype interaction included in the model were non-significant for chest girth at all the ages. The least squares means of chest girth (Table-2) revealed significant breed differences at each of age; Sirohi kids measuring significantly higher than the Barbari kids. The least squares means for genotype AB was found to be superior to BB for chest girth at all the ages. However, the difference was non-significant.

**Table-1:** Least squares analysis of variance (MS) for chest and paunch girth (cm) from birth to 180 days of age.

Effects	Chest Girth				Paunch Girth			
	
	Breed	Genotype	Breed×genotype	Error	Breed	Genotype	Breed×Genotype	Error
At birth	118.7 (1)	0.03 (1)	2.281 (1)	2.49 (76)	121.86 (1)	0.04 (1)	2.58 (1)	2.69 (76)
1^st^ week	196.40 (1)	1.50 (1)	2.59 (1)	3.63 (76)	202.94 (1)	1.69 (1)	2.67 (1)	3.81 (76)
2^nd^ week	264.44 (1)	2.77 (1)	5.78 (1)	4.77 (76)	259.45 (1)	2.86 (1)	6.55 (1)	4.88 (76)
3^rd^ week	360.53 (1)	8.90 (1)	4.56 (1)	6.55 (76)	356.34 (1)	8.87 (1)	4.27 (1)	6.67 (76)
4^th^ week	458.55 (1)	14.61 (1)	6.61 (1)	8.47 (76)	462.19 (1)	14.79 (1)	6.83 (1)	8.36 (76)
60 days	488.14 (1)	41.00 (1)	15.24 (1)	18.98 (66)	461.95 (1)	42.37 (1)	15.84 (1)	18.85 (66)
90 days	518.45 (1)	80.72 (1)	7.80 (1)	22.28 (60)	492.30 (1)	81.36 (1)	8.08 (1)	22.26 (60)
180 days	316.87 (1)	57.96 (1)	4.56 (1)	31.75 (21)	287.37 (1)	57.27 (1)	4.21 (1)	32.13 (21)

Significant (*p<0.01*), SV=Source of variation, df=degree of freedom, MS=Mean Sum of squares, Figure in parenthesis is the df for the respective source of variation

**Table-2:** Least squares means for chest girth (cm) from birth to 180 days of age.

Effects	Breed		Genotype		BxG interaction				Overall mean
		
	Sirohi (B_1_)	Barbari (B_2_)	AB (G_1_)	BB (G_2_)	B_1_G_1_	B_1_G_2_	B_2_G_1_	B_2_G_2_	
At birth	29.53^b^±0.32 (40)	25.88^a^±0.41 (40)	27.74±0.19 (69)	27.67±0.49 (11)	29.75±0.59 (33)	29.31±0.27 (7)	26.17±0.26 (36)	25.60±0.79 (4)	27.71±0.26 (80)
1^st^ week	31.70^b^±0.39 (40)	27.00^a^±0.50 (40)	29.56±0.22 (69)	29.14±0.59 (11)	31.77±0.72 (33)	31.64±0.33 (7)	27.47±0.31 (36)	26.52±0.95 (4)	29.35±0.31 (80)
2^nd^ week	33.51^b^±0.45 (40)	28.05^a^±0.57 (40)	31.06±0.26 (69)	30.50±0.68 (11)	33.64±0.82 (33)	33.39±0.38 (7)	28.74±0.36 (36)	27.37±1.09 (4)	30.78±0.36 (80)
3^rd^ week	35.18^b^±0.53 (40)	28.80^a^±0.67 (40)	32.49±0.30 (69)	31.49±0.80 (11)	35.32±0.44 (33)	35.04±0.96 (7)	29.66±0.42 (36)	27.95±1.27 (4)	31.99±0.42 (80)
4^th^ week	36.73^b^±0.60 (40)	29.54^a^±0.76 (40)	33.78±0.35 (69)	32.50±0.91 (11)	36.94±0.50 (33)	36.52±1.10 (7)	30.62±0.48 (36)	28.47±1.45 (4)	33.14±0.48 (80)
60 days	40.68^b^±0.90 (40)	33.17^a^±1.17 (30)	38.01±0.57 (59)	35.84±1.36 (11)	41.10±0.75 (33)	40.25±1.64 (7)	34.92±0.85 (26)	31.42±2.17 (4)	36.92±0.74 (70)
90 days	43.78^b^±0.99 (36)	35.99^a^±1.27 (28)	41.42±0.65 (53)	38.35±1.47 (11)	44.84±0.87 (29)	42.72±1.78 (7)	38.00±0.96 (24)	33.97±2.36 (4)	39.89±0.80 (64)
180 days	54.25^b^±1.99 (9)	46.20^a^±1.62 (16)	51.92±1.40 (18)	48.45±2.15 (7)	55.50±2.30 (6)	53.00±3.25 (3)	48.35±1.62 (12)	43.90±2.81 (4)	50.18±1.28 (25)

a,b Means of a trait under a particular effect with different superscripts differ significantly, Figures in parentheses are number of observations

Present findings are in agreement with the findings of [14]. They have also reported non-significant differences for chest girth at 2 months age between different genotypes involving single nucleotide polymorphisms at different regions of GH genes in Sangamneri breed of goat.

However, contrary to the present findings, [9] in Boer goat bucks reported significant effect of genotype on chest girth at birth and weaning; [15] in Nanjiang Huang goat found significant effect of genotype on chest girth at 2 months of age and [16] in Guanzhong goat with IGF-I gene (a gene similar to GH gene in its effect) polymorphism reported significant (p<0.05) effect of genotype on chest circumference. The least squares analysis of variance (Table-1) conducted to analyze association of polymorphic variants of GH gene with paunch girth at birth to 180 days of age evinced highly significant effect (p<0.01) of breed on paunch girth at each stage. The effect of other factors, i.e. genotype and breed × genotype interaction included in the model were non-significant for paunch girth at all the ages. The increase in estimated error from 4th week onwards might be due to decline in animal numbers over the period, i.e. variability in the data. In addition, sampling fluctuations might also have contributed to the differences in allelic frequencies in different breeds and populations. The least squares means of paunch girth (Table-3) revealed significant breed differences at each of age; the kids of Sirohi kids measuring significantly higher than the Barbari kids. Although the least squares means for AB and BB genotypes did not differed significantly at any stage, genotype AB was constantly superior over all the intervals for paunch girth.

**Table-3:** Least squares means for paunch girth (cm) from birth to 180 days of age.

Effects	Breed		Genotype		B×G interaction				Overall mean
		
	Sirohi (B_1_)	Barbari (B_2_)	AB (G_1_)	BB (G_2_)	B_1_G_1_	B_1_G_2_	B_2_G_1_	B_2_G_2_	
At birth	30.05^b^±0.34 (40)	26.32^a^±0.43 (40)	28.21±0.19 (69)	28.14±0.51 (11)	30.27±0.62 (33)	29.80±0.28 (7)	26.63±0.27 (36)	26.02±0.82 (4)	28.18±0.27 (80)
1^st^ week	32.40^b^±0.40 (40)	27.61^a^±0.51 (40)	30.22±0.23 (69)	29.79±0.61 (11)	32.45±0.73 (33)	32.34±0.34 (7)	28.11±0.32 (36)	27.12±0.97 (4)	28.18±0.27 (80)
2^nd^ week	34.19^b^±0.45 (40)	28.78^a^±0.58 (40)	31.77±0.26 (69)	31.20±0.69 (11)	34.34±0.83 (33)	34.05±0.38 (7)	29.50±0.36 (36)	28.07±1.10 (4)	31.49±0.37 (80)
3^rd^ week	36.01^b^±0.53 (40)	29.67^a^±0.68 (40)	33.34±0.31 (69)	32.34±0.80 (11)	36.16±0.44 (33)	35.85±0.97 (7)	30.51±0.43 (36)	28.82±1.29 (4)	32.84±0.43 (80)
4^th^ week	37.67^b^±0.60 (40)	30.45^a^±0.76 (40)	34.71±0.34 (69)	33.42±0.90 (11)	37.88±0.50 (33)	37.47±1.09 (7)	31.54±0.48 (36)	29.37±1.44 (4)	34.06±0.48 (80)
60 days	41.55^b^±0.90 (40)	34.25^a^±1.16 (30)	39.01±0.56 (59)	36.80±1.36 (11)	41.98±0.75 (33)	41.12±1.64 (7)	36.03±0.85 (26)	32.47±2.17 (4)	37.90±0.73 (70)
90 days	44.72^b^±0.99 (36)	37.13^a^±1.27 (28)	42.47±0.64 (53)	39.38±1.47 (11)	45.78±0.87 (29)	43.67±1.77 (7)	39.16±0.96 (24)	35.10±2.35 (4)	40.93±0.80 (64)
180 days	55.15^b^±2.00 (9)	47.42^a^±1.63 (16)	53.01±1.41 (18)	49.56±2.16 (7)	56.41±2.31 (6)	53.90±3.27 (3)	49.61±1.63 (12)	45.22±2.83 (4)	51.28±1.29 (25)

a,b Means of a trait under a particular effect with different superscripts differ significantly, Figures in parentheses are number of observations

The present findings are in agreement with the reports of [14] in Sangamneri goat breed where association of single nucleotide polymorphism with paunch girth was reported to be non-significant for different regions of GH gene.

It is evident from the least squares analysis that the measures for growth traits undertaken in the present study were significantly higher in Sirohi as compared to Barbari breed. The different genotypes of Sirohi breed also fared well though non-significantly in comparison to its Barbari counterpart. The probable reason for the present outcome may be attributed to the genetic makeup of these breeds. The differences in the performance of various genotypes might be due to differential expression of genes in different environments as a result of genotype-environment interaction, which influences animal’s physiology.

## Conclusion

The PCR restriction fragment length polymorphism technique used in this experiment proved to be an appropriate technique in screening samples for polymorphism with respect to exon 2 and exon 3 region of GH gene. The highly significant Chi-square values for both Sirohi and Barbari breeds revealed that none of the breeds was in Hardy–Weinberg equilibrium for this region of GH gene. Statistical analysis of growth traits with the genotype could not establish any significant association from birth to 180 days of age in both the breeds but comparatively higher body weights and body measurements were observed for AB genotype in both the breeds. However, as the study was based on a limited sample size, further study on larger sample is suggested.

## Authors’ Contribution

PPS carried out the experiment. SST designed the experiment, guided during the experiment. MST and AK helped in the analysis of data, drafted and revised the manuscript. All authors read and approved the final manuscript.
